# Liraglutide Reduces Both Atherosclerosis and Kidney Inflammation in Moderately Uremic LDLr-/- Mice

**DOI:** 10.1371/journal.pone.0168396

**Published:** 2016-12-16

**Authors:** Line S. Bisgaard, Markus H. Bosteen, Lisbeth N. Fink, Charlotte M. Sørensen, Alexander Rosendahl, Christina K. Mogensen, Salka E. Rasmussen, Bidda Rolin, Lars B. Nielsen, Tanja X. Pedersen

**Affiliations:** 1 Department of Biomedical Sciences, University of Copenhagen, Copenhagen, Denmark; 2 Global Research, Novo Nordisk, Måløv, Denmark; 3 Department of Clinical Biochemistry, Copenhagen University Hospital Rigshospitalet, Copenhagen, Denmark; University Medical Center Utrecht, NETHERLANDS

## Abstract

Chronic kidney disease (CKD) leads to uremia. CKD is characterized by a gradual increase in kidney fibrosis and loss of kidney function, which is associated with a progressive increase in risk of atherosclerosis and cardiovascular death. To prevent progression of both kidney fibrosis and atherosclerosis in uremic settings, insight into new treatment options with effects on both parameters is warranted. The GLP-1 analogue liraglutide improves glucose homeostasis, and is approved for treatment of type 2 diabetes. Animal studies suggest that GLP-1 also dampens inflammation and atherosclerosis. Our aim was to examine effects of liraglutide on kidney fibrosis and atherosclerosis in a mouse model of moderate uremia (5/6 nephrectomy (NX)). Uremic (n = 29) and sham-operated (n = 14) atherosclerosis-prone low density lipoprotein receptor knockout mice were treated with liraglutide (1000 μg/kg, s.c. once daily) or vehicle for 13 weeks. As expected, uremia increased aortic atherosclerosis. In the remnant kidneys from NX mice, flow cytometry revealed an increase in the number of monocyte-like cells (CD68^+^F4/80^-^), CD4^+^, and CD8^+^ T-cells, suggesting that moderate uremia induced kidney inflammation. Furthermore, markers of fibrosis (i.e. Col1a1 and Col3a1) were upregulated, and histological examinations showed increased glomerular diameter in NX mice. Importantly, liraglutide treatment attenuated atherosclerosis (~40%, p < 0.05) and reduced kidney inflammation in NX mice. There was no effect of liraglutide on expression of fibrosis markers and/or kidney histology. This study suggests that liraglutide has beneficial effects in a mouse model of moderate uremia by reducing atherosclerosis and attenuating kidney inflammation.

## Introduction

Chronic kidney disease (CKD) leads to uremia, and is one of the strongest known risk factors for cardiovascular death (CVD) [1, 2]. Presently, up to 10% of the general population are at increased risk of CVD due to decreased kidney function [3]. This number is believed to rise in the coming years due to the increase in patients with type 2 diabetes. Thus, long standing diabetes is a risk factor for CKD [3].

CKD is characterized by a detrimental, self-reinforcing, process in which loss of kidney function and subsequent kidney inflammation and fibrosis promotes disease progression to end stage renal disease (ESRD) and ultimately death [4, 5]. Although kidney fibrosis is believed to be driven by aberrant inflammatory processes, the exact mechanisms behind the progression from CKD to ESRD are not fully understood. Currently, there are no treatment options available to lower kidney fibrosis [5, 6]. There is therefore a huge need for new treatments aimed at attenuating fibrosis.

One of the main reasons for the increased risk of CVD in patients with CKD is acceleration of atherosclerosis. Current treatment modalities aimed at reducing atherosclerosis are not sufficiently effect-full in patients with reduced kidney function, and new treatment options are therefore warranted. To investigate mechanisms accelerating atherosclerosis in uremia, we—and others—have induced moderate uremia by 5/6 nephrectomy (NX) in hyperlipidemic mice and shown that this increases atherosclerosis [7–9]. In normocholesterolemic rats and mice, the NX model has also been applied to study kidney inflammation and fibrosis in moderate uremia [10–13]. Studies investigating the effects on kidney inflammation and fibrosis in a hypercholesterolemic setting are, however, highly warranted.

GLP-1 is mainly known for its role in glucose metabolism and appetite regulation, and GLP-1 based therapies are approved for treatment of type 2 diabetes and obesity. A number of studies suggest that GLP-1 has additional benefits. Thus, GLP-1 and GLP-1 analogues have been shown to have anti-inflammatory properties in humans [14] and treatment with GLP-1 and GLP-1 analogues has decreased atherosclerosis in mouse models [15–17]. Moreover, previous studies have showed protective effects of GLP-1 analogues by attenuating albuminuria and hyperfiltration in animal models of diabetes [18, 19].

The aim of the present work was to investigate whether induction of moderate uremia in atherosclerosis-prone hyperlipidemic LDLr-/- mice induced not only atherosclerosis, but also affected the renal phenotype of inflammation and fibrosis. If so, we wanted to address whether the GLP-1 analogue liraglutide could attenuate these effects in uremic settings.

## Results

### Uremia accelerates atherosclerosis and kidney fibrosis in LDLr-/- mice (Study 1)

To study the effects of uremia on atherosclerosis, kidney inflammation and fibrosis, moderate uremia was induced by 5/6 NX in LDLr-/- mice. Uremia was induced in 13 weeks old female LDLr-/- mice (NX) and LDLr-/- mice were sham-operated as controls (SHAM) (see study outline in S1 Fig). Seven weeks after induction of uremia, all mice received a cholesterol rich diet (0.3% cholesterol and 4.25% fat) for 9 weeks. The study was terminated 16 weeks after induction of uremia. At this time point, plasma analyses indicated that NX induced a moderate increase in plasma urea (~2.5 fold, P<0.0001) and creatinine (~1.5 fold, P<0.0001) concentrations (S1 Table).

**Fig 1:**
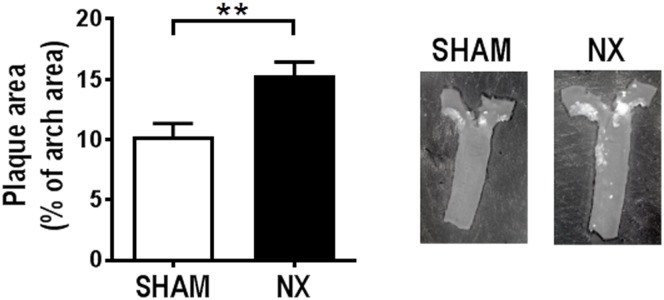
Uremia accelerates atherosclerosis in LDLr-/- mice. Uremic (NX; n = 10) and control (SHAM; n = 10) LDLr-/- mice were put on a western type diet for 9 weeks after induction of uremia. Atherosclerosis was determined as the relative plaque area in % of the total aortic arch area. Depicted values are mean±SEM. *p<0.05 as determined by unpaired students t-test.

**Fig 2:**
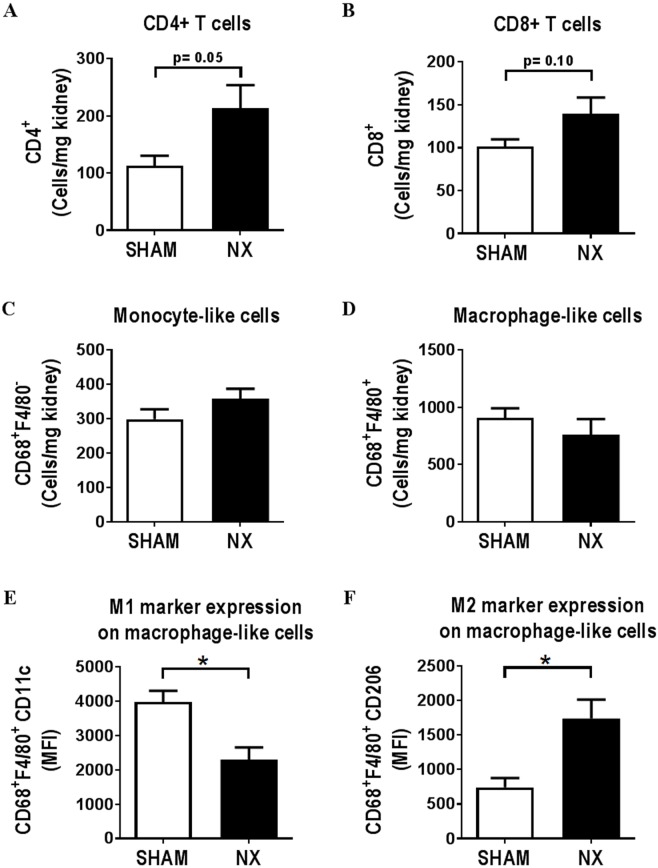
Immune cell composition is changed in kidneys from uremic LDLr-/- mice. Sixteen weeks after induction of uremia, flow cytometry was performed on kidneys from control (SHAM) and uremic (NX) LDLr-/- mice (n = 5 mice/group). The number of CD4^+^ T-cells (**A**), CD8^+^ T-cells (**B**), monocyte-like (CD68^+^F4/80^-^) and macrophage-like (CD68^+^F4/80^+^) cells (**C and D**) relative to kidney weight is depicted. On macrophage-like cells, median fluorescent intensity (MFI) for the M1 marker CD11c (**E**) or the M2 marker CD206 (**F**) was detected. Depicted values are mean±SEM. *p<0.05 as determined by unpaired students t-test.

Aortic arch atherosclerosis was significantly increased in NX as compared to SHAM mice (Fig 1). Flow cytometry analyses of the remnant kidney from NX mice and an anatomically comparable kidney piece from SHAM mice revealed a reduction of endothelial and distal tubular epithelial cells (DTECs) in the remnant kidneys from NX mice compared to SHAM mice. The number of proximal epithelial cells (PTECs) was not affected (S2 Fig). Leukocyte numbers were identical in SHAM and NX mice (S3 Fig) and based on the number of neutrophils, there was no sign of acute inflammation in the kidneys (data not shown). However, there was a trend, albeit not statistically significant, towards an increased number of CD4^+^ T-cells in the remnant kidney from NX as compared to SHAM mice (p = 0.05), and a similar not statistically significant trend for CD8^+^ T-cells (p = 0.10) (Fig 2A and 2B). Neither monocyte-like (CD68^+^F4/80^-^) nor macrophage-like (CD68^+^F4/80^+^) cell numbers were affected by NX (Fig 2C and 2D). Of interest, in the macrophage-like cell population, there was a shift towards a pro-fibrotic M2-like phenotype with increased expression of the M2 marker CD206 and decreased expression of the M1 marker CD11c (Fig 2E and 2F).

Gene expression analyses revealed an upregulation of fibrosis-related genes in the remnant kidney of NX as compared to SHAM mice (Table 1). Thus, mRNA expression of Col1a1, Col3a1, and Fn1 was significantly increased ~2-, ~2-, and ~4-fold, respectively. Moreover, there was an upregulation of the kidney injury marker NGAL, while Wt1, a marker for podocytes, was downregulated, altogether indicating kidney injury (Table 1). Notably, of the 8 inflammatory genes investigated, only Ccl12 was significantly upregulated by NX (~2.4-fold, p<0.05) (Table 1).

**Table 1:** Gene expression depicted as ΔCT and fold change relative to control (SHAM). Results are shown as (mean±SEM).

Gene name	SHAM	NX	NX rel to SHAM
	ΔCT	ΔCT	mean fold change
**Fibrosis**			
Col1a1	5.74±0.10	4.75±0.19	2.04±0.34*
Col1a2	5.26±0.12	4.88±0.17	1.31±0.17
Col3a1	5.99±0.14	4.95±0.18	2.08±0.31*
Ctgf	2.26±0.07	2.36±0.19	0.96±0.12
Fn1	5.39±0.11	3.51±0.30	4.04±0.98*
Serpine1	7.74±0.14	7.26±0.14	1.36±0.14*
Tgfb1	6.71±0.08	6.77±0.18	0.99±0.14
Tgfb2	7.90±0.11	7.73±0.16	1.13±0.11
Tgfb3	9.46±0.12	8.78±0.16	1.63±0.23*
Snai1	8.17±0.26	9.11±0.25	0.47±0.07*
Vim	4.68±0.19	4.12±0.20	1.42±0.20
Pdgfb	5.82±0.10	5.93±0.16	0.94±0.12
Pdgfrb	4.53±0.09	4.87±0.11	0.79±0.06*
**Adhesion molecules**			
Cdh1	3.72±0.06	4.01±0.14	0.83±0.09*
Cdh5	4.60±0.09	4.56±0.12	1.03±0.09
Itga1	4.60±0.12	4.65±0.10	0.96±0.07
Itga3	6.22±0.06	6.66±0.08	0.74±0.04*
Itga5	7.84±0.12	8.06±0.15	0.86±0.10
**Extracellular matrix**			
Hspg2	4.95±0.13	5.10±0.06	0.88±0.03
Lamc1	6.60±0.11	6.75±0.10	0.89±0.06
Mmp2	7.99±0.13	7.33±0.21	1.61±0.23*
Timp1	11.28±0.12	10.15±0.14	2.18±0.20*
Timp2	4.05±0.09	4.00±0.13	1.04±0.10
**Kidney injury**			
Cdkn1a	7.53±0.16	7.16±0.19	1.27±0.16
Havcr1	10.51±0.25	9.93±0.37	1.54±0.39
Lcn2 (NGAL)	7.50±0.14	4.99±0.21	5.76±0.88*
Wt1	7.43±0.11	8.09±0.08	0.62±0.03*
Nphs1	6.89±0.09	7.33±0.12	0.74±0.07*
**Inflammation**			
Tlr2	8.19±0.12	7.79±0.20	1.35±0.22
Tnf	11.85±0.16	11.29±0.34	1.62±0.49
Ccl12	9.98±0.19	8.70±0.25	2.42±0.43*
Ccl2	9.49±0.24	8.84±0.26	1.52±0.34
Ccl5	8.43±0.28	8.42±0.38	0.99±0.23
Cxcl15	14.78±0.55	13.55±0.38	2.08±0.68
Icam1	7.95±0.17	8.12±0.24	0.90±0.16
Vcam1	7.08±0.07	6.68±0.24	1.39±0.23
**BMP system**			
Grem1	12.53±0.20	9.86±0.34	6.66±1.26*
Kcp	9.46±0.10	9.29±0.16	1.14±0.13
**Hypoxia**			
Hif1a	2.82±0.05	2.78±0.08	1.03±0.06
Nos2	10.84±0.11	10.87±0.27	1.05±0.23
Nos3	7.90±0.09	8.32±0.13	0.75±0.07*
**Rage**			
Ager	10.56±0.11	10.67±0.10	0.91±0.06
**RAAS**			
Ace	4.10±0.13	4.32±0.13	0.84±0.08
Agt	4.78±0.11	4.15±0.17	1.56±0.22*

* p<0.05 relative to SHAM as determined by unpaired students-t test.

n = 6 (NX) and 10 (SHAM). Col1a1: Collagen, type I, alpha 1, Col1a2: Collagen, type I, alpha 2, Col3a1: Collagen, type III, alpha 1, Ctgf: Connective tissue growth factor, Fn1: Fibronectin 1, Tgfb1: Transforming growth factor beta 1, Tgfb2: Transforming growth factor beta 2, Tgfb3: Transforming growth factor beta 3, Snai1: Snail family zinc finger 1, Vim: Vimentin, Pdgfb: Platelet-derived growth factor subunit B, Pdgfrb: Platelet derived growth factor receptor beta, Cdh1: Cadherin 1, Cdh5: Cadherin 5, Itga1: Integrin subunit alpha 1, Itga3: Integrin subunit alpha 3, Itga5: Integrin subunit alpha 5, Hspg2: Heparan sulfate proteoglycan 2, Lamc1: Laminin subunit gamma 1, Mmp2: Matrix metallopeptidase 2, Timp1: Tissue inhibitor of metalloproteinase 1, Timp2: Tissue inhibitor of metalloproteinase 2, Cdkn1a: Cyclin-dependent kinase inhibitor 1a, Havrc1: Hepatitis A virus cellular receptor 1, Lcn2: Lipocalin-2, Wt1: Wilms tumor 1, Nphs1: Nephrin, Ccl2: Chemokine (C-C motif) ligand 2, Tlr2: Toll like receptor 2, Tnf: Tumor necrosis factor, Ccl12: Chemokine (C-C motif) ligand 12, Ccl2: Chemokine (C-C motif) ligand 2, Ccl5: Chemokine (C-C motif) ligand 5, Cxcl15: Chemokine (C-X-C motif) ligand 15, Icam1: Intercellular adhesion molecule 1, Vcam1: Vascular cell adhesion molecule 1, Grem1: Gremlin 1, Kcp: Kielin/chordin-like protein, Hif1a: Hypoxia inducible factor 1, alpha subunit, Nos2: Nitric oxide synthase 2, Nos3: Nitric oxide synthase 3, Ager: Advanced glycosylation end product-specific receptor, Ace: Angiotensin I converting enzyme, Agt: Angiotensinogen

Overall, the results obtained in Study 1 suggested that 5/6 NX in LDLr-/- mice provides a suitable model for investigations of both atherosclerosis and the renal phenotype of inflammation and fibrosis, in relation to moderate uremia.

### Liraglutide induces a modest weight reduction, but does not affect plasma cholesterol concentrations in uremic mice (Study 2)

The GLP-analogue liraglutide has been shown to have beneficial effects on both atherosclerosis and diabetic kidney disease in animal models (15, 19). To address whether liraglutide could affect atherosclerosis and kidney damage in uremic LDLr-/- mice, we induced moderate uremia by NX in 11–12 weeks old LDLr-/- mice and included SHAM-operated controls (Study 2) (see study outline in S1 Fig). Prior to initiation of liraglutide treatment, plasma urea and creatinine concentrations were increased ~3-fold (S2 Table). NX mice were divided into 2 separate groups matched by weight, cholesterol, and urea concentrations. Two weeks after induction of uremia, injections of liraglutide (once daily) or vehicle (once daily) were initiated and maintained for 13 weeks. The dose of liraglutide was slowly increased during the first 2 weeks to minimize effects of liraglutide on food intake. When the full dose was reached (1000 μg/kg), mice were switched to cholesterol enriched diet. The study was terminated 15 weeks after induction of uremia. Measurements of plasma liraglutide concentrations during the study showed that liraglutide did not accumulate in the blood of NX mice (liraglutide concentration in plasma after 2 weeks: 191±21 nM; 8 weeks: 252±20 nM; and at termination: 250±25nM; p = ns). Furthermore, liraglutide treatment did not affect plasma glucose concentrations (S4 Fig).

At termination, liraglutide had not affected plasma urea, creatinine, or cholesterol concentrations (S2 Table). There was a small, yet statistically significant, weight difference between the NX LIRA group and the NX control group at study termination (S2 Table). Also, a graphical depiction suggested a lower accumulated food intake in the NX LIRA group (S5 Fig). Indeed, this was significant if the food intake was estimated as food intake per day per group normalized to the number of mice in each group (SHAM: ~2.5 g/day, NX: ~2.7 g/day, NX LIRA: ~2.4 g/day, p = 0.002, one-way ANOVA).

### Liraglutide attenuates atherosclerosis in uremic mice but has only minor effects on vascular inflammation

**Fig 3:**
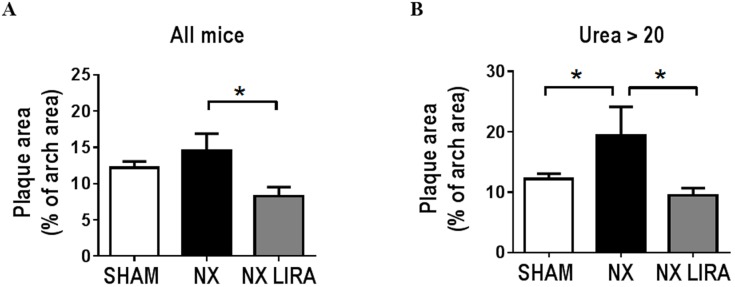
Liraglutide attenuates atherosclerosis in uremic LDLr-/- mice. Uremic LDLr-/- mice were treated with vehicle (NX; n = 14) or liraglutide (NX LIRA; n = 15) and sham operated control LDLr-/-mice were treated with vehicle (SHAM; n = 14). After 11 weeks of treatment with full dose (1000 μg/kg), atherosclerosis was quantified as the relative plaque area in % of the total aortic arch area in all mice (**A**) and in NX mice with urea levels >20 (**B**). Depicted values are mean±SEM. *p<0.05 as determined by 1-way ANOVA followed by Sidak’s multiple comparisons post-test. n = 14–15 mice per group.

Atherosclerosis was assessed in the aortic arch *en face*. Unexpectedly, there was no statistically significant increase in plaque formation in NX mice compared to SHAM mice (Fig 3A). However, when examining ‘mildly uremic’ (plasma urea<20 mM) as compared to ‘moderately uremic’ (plasma urea>20 mM) mice, we detected a 1.5 fold uremia-mediated acceleration of atherosclerosis in ‘moderately uremic’ as compared to SHAM mice (p = 0.02, Fig 3B). Importantly, liraglutide significantly attenuated plaque formation regardless of whether the statistical analyses included all uremic mice or only ‘moderately uremic’ mice (Fig 3A and 3B).

To address whether the effect of liraglutide was paralleled by changes in vascular inflammation, aortic gene expression analyses and flow analyses of aortic immune cells were performed. Although there was a non-significant trend towards lower leukocyte numbers in liraglutide treated mice in the aortic wall (S6A Fig), there were no significant changes in the myeloid or lymphoid composition, or in macrophage polarization markers (S6B–S6D Fig). Also, aortic mRNA expression of the inflammatory markers MCP-1, VCAM-1, and iNOS was not affected by liraglutide treatment (data not shown).

NX increased the plasma concentration of both pro- (OPN, IFNγ and TNFα) and anti-inflammatory cytokines (IL-10). These analyses did not reveal effects of liraglutide on plasma cytokine concentrations in NX mice (S3 Table).

### Inflammatory changes induced by NX in the kidney is attenuated by liraglutide

To determine effects of liraglutide on kidney inflammation and fibrosis, we used a combination of flow cytometry, gene expression, and histology. The number of endothelial cells, DTECs, and PTECs was decreased in NX as compared to SHAM mice. Liraglutide had no effect on these parameters in NX mice (S7 Fig).

**Fig 4:**
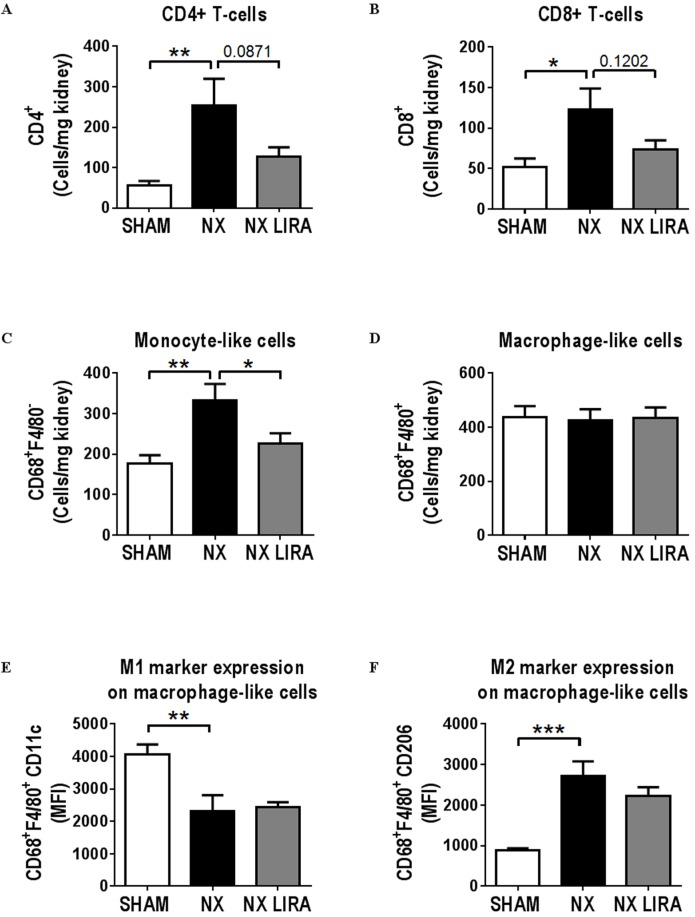
Liraglutide attenuates NX mediated kidney inflammation in LDLr-/- mice. A-F: flow cytometry analysis of kidneys from control (SHAM; n = 7), uremic (NX; n = 7) or liraglutide treated uremic (NX LIRA; n = 7) LDLr-/- mice showing the number of CD4^+^ T-cells (**A**), CD8^+^ T-cells (**B**), monocyte-like (CD68^+^F4/80^-^) and macrophage-like (CD68^+^F4/80^+^) cells (**C** and **D**) relative to kidney weight. On macrophage-like cells, median fluorescent intensity (MFI) for the M1 marker CD11c (**E**) or the M2 marker CD206 (**F**) was detected. Depicted values are mean±SEM. *p<0.05, **p<0.01, ***p<0.005 as determined by 1-way ANOVA followed by Sidak’s multiple comparisons post-test. n = 6 mice per group.

The inflammatory profile of the NX remnant kidney was roughly similar to that seen in Study 1; i.e. absence of acute inflammation as determined by the number of neutrophils (data not shown), increased CD4^+^ and CD8^+^ T-cells (Fig 4A and 4B), and identical number of macrophage-like cells. Also, there was a skewing of the macrophage-like cells towards a more pro-fibrotic M2 like phenotype (Cd11c^low^CD206^high^) in both studies (Fig 4D–4F). In addition, there was increased numbers of total leukocytes (S8 Fig) and monocyte-like cells (Fig 4C). Interestingly, liraglutide treatment reduced the number of monocyte-like cells (Fig 4C). Furthermore, there was a non-significant attenuation of leukocyte, CD4^+^T-cell, and CD8^+^ T-cell, infiltration after liraglutide treatment (S8 Fig, Fig 4A and 4B), while expression of neither the M1 nor the M2 marker on macrophage-like cells was altered (Fig 4E and 4F).

**Fig 5:**
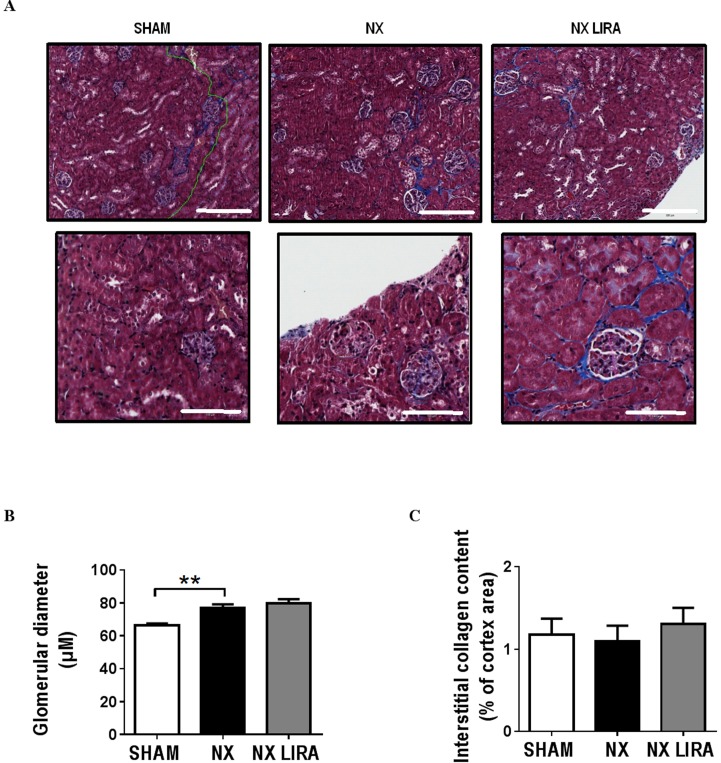
Uremia increases glomerular size, but not cortical collagen content in LDLr-/- mice. Representative pictures of kidney sections from control (SHAM), uremic (NX) and liraglutide treated uremic (NX LIRA) mice stained with Massons trichrome (**A**). Scale bar = 200 μm for top row and 100 μm for bottom row. Glomerular diameters were measured (**B**) (25–49 glomeruli were assessed per kidney, n = 7–8 in each group) and collagen deposition in the kidney cortex was quantified (**C**) (n = 5–7 in each group) using the Visiopharm software. **p<0.01, as determined by 1-way ANOVA followed by Sidak’s multiple comparisons post-test.

Gene expression of kidney injury markers and selected key fibrosis related genes revealed changes in Col1a1, Col3a1, Fn1, Lcn2, and Havcr1 in NX compared to SHAM mice that were reminiscent of those seen in Study 1 (Table 1). There was no effect of liraglutide treatment on gene expression in NX mice (Table 2). Histological analyses of kidney sections showed an increase in glomerular size in NX as compared to SHAM mice (Fig 5A and 5B), whereas collagen deposition in the kidney cortex was not affected by NX (Fig 5A and 5C). Liraglutide did not affect these histological measures (Fig 5A–5C).

**Table 2:** Gene expression depicted as ΔCT and fold change in NX relative to control (SHAM) in column 4 and in NX LIRA relative to NX in column 5. Results are shown as mean±SEM.

Gene name	SHAM	NX	NX LIRA	NX rel. to SHAM	NX LIRA rel. to NX
	ΔCT	ΔCT	ΔCT	mean fold change	mean fold change
**Fibrosis**					
Col1a1	2.01±0.31	0.29±0.28	0.29±0.30	3.22±0.66**	1.05±0.24
Col3a1	3.77±0.18	1.72±0.11	1.68±0.15	4.02±0.30****	1.04±0.10
Fn1	2.77±0.41	0.17±0.56	0.78±0.29	7.55±2.79**	0.48±0.09
**Kidney injury**					
Cdkn1a	6.45±0.33	5.00±0.35	5.13±0.36	2.80±0.65	0.98±0.29
Havcr1	10.88±0.29	9.16±0.28	8.43±0.50	3.22±0.61*	2.25±0.75
Lcn2 (NGAL)	7.21±0.07	3.03±0.47	3.06±0.29	25.58±10.11****	0.82±0.21
Wt1	6.81±0.13	7.02±0.14	6.97±0.15	0.86±0.08	1.05±0.10
Nphs1	7.18±0.14	6.97±0.11	7.01±0.15	1.14±0.09	0.99±0.11

* p<0.05,

** p<0.01,

*** p<0.005

**** p<0.0001 relative to SHAM as determined by 1-way ANOVA followed by Sidak’s multiple comparisons post-test.

n = 7–8 mice per group. Col1a1: Collagen, type I, alpha 1, Col3a1: Collagen, type III, alpha 1, Fn1: Fibronectin 1, Cdkn1a: Cyclin-dependent kinase inhibitor 1a, Havrc1: Hepatitis A virus cellular receptor 1, Lcn2: Lipocalin-2, Wt1: Wilms tumor 1, Nphs1: Nephrin, Ccl2: chemokine (C-C motif) ligand 2.

## Discussion

In this study, we have established that hypercholesterolemic 5/6 nephrectomized (NX) mice can be used to study both uremia mediated acceleration of atherosclerosis and a renal phenotype of inflammation and early fibrotic changes. We show that treatment with the GLP-1 analogue liraglutide attenuates atherosclerosis and infiltration of monocyte-like cells in the kidney in uremic mice. Moreover, we find a tendency to less infiltration of CD4^+^ and CD8^+^ T cells in kidneys from liraglutide treated uremic mice.

### NX effects on the (remnant) kidney

Uremia promotes progressive kidney injury and fibrosis. Inflammation is central in the fibrotic process, since an aberrant inflammatory stimulus drives the activation of fibroblasts leading to extracellular matrix deposition. In this study, remnant kidneys from 5/6 NX LDLr-/- mice were carefully examined to establish the inflammatory and fibrotic status in moderate uremia. We established that this model is characterized by inflammatory cell infiltration (i.e. more CD4^+^ and CD8^+^ T cells as well as a marked shift towards more pro-fibrotic M2-like macrophages) in NX compared to SHAM mice. Moreover, induction of uremia increased expression of fibrosis-related genes, most importantly Col1a1, Col3a1, and Fn1. This apparent pro-fibrotic environment in the NX mice did not lead to increased fibrosis as determined by collagen deposition in the kidney cortex, though we detected an increased glomerular diameter suggesting a response to the inflammatory environment [20]. This latter observation may also simply reflect a compensatory increase to maintain glomerular filtration after removal of 5/6 of the kidney tissue. One explanation for the lack of induction of fibrosis may be the use of C57Bl6/j mice. While 5/6 NX in both rats and FVB/N mice results in both glomerulosclerosis and tubulointerstitial fibrosis [21, 22], the C57Bl6/j strain, which is the only background strain that readily develops atherosclerosis, is more resistant to kidney fibrosis [21, 23]. Similarly, a recent study by Ghosh et al also did not detect increased fibrosis in this model [24]. Furthermore, previous studies in our group [8, 25, 26] have established that kidney function after 5/6 NX does not deteriorate progressively with time (up to 1 year) in C57Bl6/J mice. It is therefore possible that 5/6 NX on a C57Bl6/J background merely reflect early stages of kidney injury, incl. inflammatory cell infiltration, ‘fibrotic’ gene expression, and increased glomerular diameter.

To our knowledge, we are amongst the first to document the effects of NX on the inflammatory state in remnant kidneys from hypercholesterolemic mice. Such a setting is highly relevant since it enables examination of effects on atherosclerosis (which requires hypercholesterolemia) and kidney damage in the same model.

### Liraglutide effects in whole animals

Since GLP-1 analogues have been shown to have beneficial effects on both classical atherosclerosis and kidney injury, we analyzed whether there might be effects of liraglutide on both atherogenesis and kidney damage in NX mice. Although there was a tendency to lower creatinine levels in NX mice treated with liraglutide, treatment did not affect plasma biochemistry; including urea, Ca^2+^, phosphate, and cholesterol levels. Also, we did not detect accumulation of liraglutide in NX mice, despite the relatively high dose given. Similar to what has been found in a previous study addressing plaque formation in ApoE-/- mice, we found that liraglutide treatment resulted in a slightly lower body weight (15). Furthermore, liraglutide treatment resulted in a minor reduction in food intake. However, we did not detect any difference in plasma cholesterol concentrations, and it is therefore unlikely that the attenuating effects of liraglutide on uremic atherosclerosis are caused by the slight reduction in food intake.

### Liraglutide effects on uremic atherosclerosis

We show that treatment with a GLP-1 analogue significantly attenuates atherosclerosis in a uremic setting. Previously, mouse studies have shown that native GLP-1 and GLP-1 analogues attenuate atherosclerosis [15–17], and it has been hypothesized that this might be caused by decreased vascular inflammation [15, 17, 27, 28]. Thus, it has been suggested that the effect of GLP-1 analogues on atherosclerosis is mediated via decreased macrophage influx [17, 27]. Despite a statistically non-significant trend towards lower leukocyte numbers in liraglutide as compared to saline treated NX mice, we did not see effects of liraglutide on myeloid or lymphoid cell composition in the aorta. Furthermore, liraglutide treatment does not affect plasma cytokine levels or inflammatory gene expression in the aorta for the parameters we have investigated. Taken together, this suggests that the effect of liraglutide on uremic atherosclerosis is not driven by attenuation of vascular inflammation.

Alternatively, based on the observation that GLP-1 or a GLP-1 analogue reduces cholesterol accumulation in macrophages, it could be speculated that liraglutide treatment results in less uptake or increased efflux of cholesterol in macrophages and hence attenuated foam cell formation [16, 29]. Also, GLP-1 receptors are present on vascular smooth muscle cells (VSMCs). VSMCs likely play an important role in plaque progression and perhaps even more so in uremia. Thus, up to 30% of all lesion cells are derived from VSMCs [30] and uremia promotes a phenotypic switch towards a pro-atherogenic aortic VSMC phenotype [31, 32] and unpublished data from our group. Thus, the present anti-atherogenic effect of liraglutide could reflect direct effects on macrophages, VSMCs, or both cell types. Further studies are needed to reveal such mechanisms.

### Liraglutide effects on the remnant kidney in NX mice

Macrophages affect kidney disease progression and fibrosis [5, 33]. Ablation of macrophages—or inhibition of macrophage influx—attenuates kidney injury and fibrosis [34–36]. Furthermore, infiltration of CD4^+^ and CD8^+^ T-cells seemingly is also important in kidney disease progression [13, 36–39], although contradictory results regarding the role of CD4+ T cells exist [40] and the exact role(s) played by inflammatory cells in kidney fibrosis is not delineated.

In the present study, NX led to a more pro-fibrotic environment in the remnant kidney, characterized by more CD4^+^ and CD8^+^ T cells, increased infiltration of monocyte-like (CD68^+^ F4/80^-^) cells and a skewing of macrophage profiles towards more M2-like, and less M1-like cells. Importantly, liraglutide tended to lower levels of CD4^+^ and CD8^+^ T cells as well as macrophage numbers in the remnant kidney after NX. In line with this observation, Kodera *et al*, reported that treatment of diabetic rats with the GLP-1 analogue exendin-4 attenuated macrophage infiltration in the kidney [19]. These authors also found that treatment with exendin-4 in NX rats resulted in reduced glomerular diameter and reduced deposition of type IV collagen [19]. Similar results have recently been shown in non-diabetic 5/6-nephrectomised rats treated with the dipeptidyl peptidase inhibitor linagliptin [41]. We did not detect effects of liraglutide on glomerular size, or expression of fibrosis markers. This was somewhat surprising, since we would have assumed that inflammation would precede an increased expression of pro-fibrotic genes and glomerular diameter—ultimately leading to kidney fibrosis. The lack of liraglutide effect could, however, be a matter of the severity of the induced kidney disease. Thus, we did not detect increased fibrosis in NX mice, despite infiltration of inflammatory cells and increased pro-fibrotic gene expression and glomerular diameter. Combined with the observation that kidney function does not get progressively worse with time in the 5/6 NX model on a C57BL6/J background, our results suggest that the LDLr-/- NX model is mimicking very early stages of kidney disease similar to mice with streptozotocin-induced diabetes [42, 43].

### Summary and conclusion

The present study suggests that 5/6 nephrectomy of hypercholesterolemic LDLr-/- mice provides the opportunity to analyze effects of a given treatment on both atherosclerosis and kidney damage. Furthermore, we show that liraglutide reduces atherosclerosis and inflammatory cell infiltration in the kidney of LDLr-/-mice in uremic settings, thus highlighting a potential additional effect of this GLP-1 analogue. The clinical importance of these findings is however unknown.

## Methods

### LDLr-/- mice: Nephrectomy, diet, and liraglutide treatment

Female LDLr^-/-^ mice (B6.129S7-Ldlrtm1Her/J, The Jackson Laboratory, Bar Harbor, USA) were housed under a 12 hour light/dark cycle with water and food ad libitum. Ten-to-thirteen weeks old mice were subjected to 5/6 nephrectomy (NX), as previously described [7, 8] to induce mild to moderate uremia. Briefly, 5/6 nephrectomy was achieved in a 2-step operation. The 2 poles of the right kidney were removed in the 1^st^ operation, while the entire left kidney was removed 2 weeks later. Control LDLr-/- mice were sham-operated. Mice where anesthetized with either a mixture of fentanyl (0.079 mg/mL), fluanisone (2.5 mg/mL), and midazolam (1.25 mg/mL) (hypnorm/dormicum) at a dose of 0.01 mL/g mouse or Zoletil (tiletamin 1.63 mg/mL, zolazepam 1.63 mg/mL, xylazin, 2.61 mg/mL, butorphanoltartrat 0.065 mg/mL) at a dose of 0.01 mL/g mouse, and analgesia was administered by subcutaneous injection of buprenorphine (0.05 mg/kg mouse) for 2–3 days. Mice were monitored closely during the surgery and in the following days.

Two studies were conducted. In study 1, mice were put on a cholate–free Western type diet with 0.3% cholesterol and 4.25% fat (D01061402, Brogaarden) 7 weeks after the 2^nd^ operation and the study was terminated 9 weeks later, i.e. 16 weeks after NX induction. In study 2, injections of either liraglutide or saline control (s.c. once daily) were initiated 2 weeks after the 2^nd^ operation and for the remainder of the study. To minimize effects of liraglutide on food intake, the liraglutide dose was slowly increased over a 2 week period until a final dose of 1000 μg/kg was reached. From the initiation of liraglutide or vehicle (20 mM phosphate, 130 mM NaCl, 0.05% tween 80, pH = 7.4) injections, respectively, food intake was measured daily by weighing the food from each cage, while the weight of each mouse was determined weekly. These weekly measurements were used to adjust the dosage of liraglutide. When the full liraglutide dose was reached (4 weeks after the 2^nd^ surgery), mice were put on the Western type diet detailed above. The study was terminated after 11 weeks on the western type diet (15 weeks after the 2^nd^ surgery). Following the 2^nd^ surgery, blood samples were collected after 1½ weeks (just before liraglutide treatment was initiated), 4 weeks (before western type diet), and 9 weeks (approximately in the middle of the diet period). In both study 1 and study 2 mice received chow diet prior to the western type diet.

In Study 1 (n = 40 mice in total), five mice died in connection with the surgical procedures, one mouse died when doing IPGTT and two mice were found dead (unknown reason). One mouse had a wound that required topical application of fucidin (2% fucidic acid) or fuciderm (0.5% fucidic acid, 0.1% w/w betamethasone). In Study 2 (n = 61 mouse in total), five mice died in relation to the surgical procedures, seven mice were euthanized due to wounds (caused by fighting and/or scratching/licking), two mice died during sampling of blood and four mice were euthanized due to humane endpoints (weight loss or general physical well-being). Three mice had wounds that required topical application of fucidin (2% fucidic acid) or fuciderm (0.5% fucidic acid, 0.1% w/w betamethasone).

### Termination of the in vivo experiments

At termination, mice were anesthetized with Zoletil (as described above), and perfused with ice-cold saline. The aortic arch was carefully dissected free from the heart and down to the 7^th^ rib, and pictures were taken for *en face* measurements. In study 2, aortas were subsequently placed in either ice-cold PBS for flow analysis or snap frozen in liquid nitrogen for RNA extraction.

The kidney/kidney remnant from all mice was carefully dissected free from the surrounding tissue, and either placed in University of Wisconsin cold-storage solution (UW solution) for flow analysis, snap frozen and stored at -80°C for RNA quantification, or fixed in 4% paraformaldehyde (PFA) for 24 hours and paraffin embedded for histology.

In study 2, the kidneys that were not used for flow cytometry were carefully cut into 2 through the midline and preserved for RNA quantification (snap frozen) or histology (embedding in Tissue-tek and frozen).

### Ethical permission

All experiments were performed according to the principles stated in the Danish law on animal experiments and were approved by the Animal Experiment’s Inspectorate, Ministry of Justice, Denmark (approval no. 2013-15-2934-00843). The investigation conforms to the Guide for the Care and Use of Laboratory Animals published by the European Parliament [EU directive 2010/63/EU].

### En face measurements of atherosclerosis

Aortas were opened longitudinally and pictures were taken with the IM50 software (Leica). Relative atherosclerotic plaque area was calculated as plaque area relative to total aortic arch surface area using the image analyses software Visiopharm (Visiopharm, Denmark).

### Plasma measurements

Blood was collected in either EDTA or heparinized microtubes and centrifuged at 4000 rpm for 10 min at 4°C. Plasma urea, creatinine, cholesterol, total calcium, and phosphate were measured with a Cobas^®^ 8000 modular analyzer series (Roche A/S). For plasma glucose concentrations, plasma samples were pooled two and two and measured with a Cobas^®^ 8000 modular analyzer series (Roche A/S).

Plasma osteopontin was measured by ELISA (Quantikine, MOST00; R&D systems), MCP-1 was measured by alphaLISA (AL509C; Perkin Elmer). TNFα, KC, IL-6, IFNγ, IL-10, and IL-5 were measured with a V-PLEX Proinflammatory Panel 1 (mouse) Kit from MSD (MESO Scale Diagnostics, K15048D). Liraglutide was measured by the assay department at Novo Nordisk using the AlphaScreen technology (Perkin Elmer, Denmark).

### RNA extraction and qPCR

In study 2, RNA was extracted form aortas using the Trizol reagent (Invitrogen). cDNA was generated using High Capacity cDNA Reverse Transcription Kit (Applied Biosystems) and analyzed with Fast SYBR ^®^ Green Master Mix on an ABI 7900 HT sequence detection system (Applied Biosystems^™^, CA, USA) using 1 ng of RNA/reaction.

RNA was extracted from kidney/kidney remnant using Trizol (Invitrogen) followed by RNeasy spin columns. Of note, both poles on the right kidney from SHAM mice were removed before RNA extraction to ensure extraction from anatomically similar pieces in NX and SHAM mice. RNA concentration was determined by Nanodrop (Thermo Scientific). In Study 1, cDNA was made from 500 ng RNA using High Capacity cDNA Reverse Transcription Kits (Applied Biosystems) and gene expression was analyzed with a Custom TaqMan^®^ Array Card (Life Technologies) using TaqMan^®^ Gene Expression Master Mix (ThermoFisher Scientific) (500 ng RNA/sample corresponding to 10 ng RNA/reaction). Assay ID for each of the genes is listed in S4 Table. In Study 2, cDNA was synthesized with SuperScript^®^ VILO^™^ cDNA Synthesis Kit (Invitrogen) and gene expression determined in duplicates using the TaqMan^®^ Fast Universal PCR Master Mix (ThermoFisher Scientific) (20 ng RNA/reaction). Assay ID for each gene can be found in S4 Table. In study 1, gene expression for target genes was normalized to the geomean of Rpl27, Rps13 and Ubc, while Rps13 was used in study 2.

### Flow cytometry analysis

Single cell suspension of aortic cells were achieved by cutting the aortas in small pieces and incubating them in an enzyme cocktail containing 450 U/mL collagenase I, 125 U/mL collagenase XI, 60U/mL hyaluronidase, and 60 U/mL DNase I in PBS for 1 hour at 37°C under slow shaking. The digested aortas were passed through a 70 μM cell strainer, centrifuged at 1200 rpm for 5 min at 4° and resuspended in flow cytometry buffer (2% heat inactivated fetal calf serum (HI FCS) and 2 mM EDTA in PBS w/o salts).

Prior to flow of kidney cells, the kidney/kidney remnant was carefully inspected and all fat etc. was removed. In kidneys from SHAM operated mice, both poles were dissected away to make sure that the kidney pieces from SHAM mice were anatomically comparable to the kidney remnants from NX mice. Kidney pieces were weighed, cut into small pieces and incubated in RPMI media (containing 5% FBS, 1% Pen/Strep, 50μM MeOH, 0,2% NaHCO_3_ 10 mM Hepes and 1 mM Sodium pyruvate) supplemented with Collagenase (2 mg/mL) and DNase (500 U/mL) for 40 min at 37°C under slow rotation. Suspensions were then mixed thoroughly and passed through a 100μM cell strainer. In Study 2, red blood cells were then lysed using cell lysis buffer (eBioscience) due to insufficient perfusion of the mice. To digest the glomerular structures, kidney suspensions were further incubated in RPMI (as described above) supplemented with 50 mg/mL Collagenase, 50 mg/mL Dispase II, 500 U DNase and 0.25% Trypsin for 15 min. at 37°C under slow rotation. The cells were then washed, centrifuged for 5 min at 1200 rpm at room temperature and resuspended in HBSS w/o salts with 2 mM EDTA and incubated for 10 min on ice. To disrupt tubular structures the suspensions were passed through a long needle (70/0.63 mm 23G, SG) 3 times, and subsequently passed through a 40 μM cell strainer. Finally, cells were centrifuged at 1200 rpm for 5 min and resuspended in flow buffer (2% heat inactivated fetal calf serum (HI FCS) and 2 mM EDTA in PBS w/o salts).

For flow cytometry of both aortas and kidneys, cells were blocked for unspecific binding with anti-CD16/CD32 (BD) and fixed and permeabilised using the Cytofix/Cytoperm solution kit (BD Biosciences). Aorta cells were surface stained with CD45 (eBioscience), CD11b (Biolegend) and CD11c (BD Biosciences), all diluted in flow cytometry buffer, and intracellularly stained for CD206 (AbdSerotic) diluted in permeabilizing buffer.

Kidney cells were surface stained with 7AAD, CD4 (BD Biosciences, San Jose, CA, USA), CD45 (eBioscience), CD45, CD31, CD8a, F4/80 (Biolegend, San Diego, Ca, USA), ENAC (Alamone, Jerusalem, Israel), CD8a, Ly6G, and Cd11c (BD- Pharmingen). Cells were intracellularly stained for CD206 and CD68 (AbDSerotec). The cellular composition was analyzed on a FACS LRSFortessa equipped with blue, red, and violet laser and data analysis was carried out using FACSDiva software (BD).

Leukocytes were defined as CD45^+^ cells. Endothelial cells were defined as CD45^-^CD31^+^ cells. Distal tubular epithelial cells were defined as CD45^-^CD31^-^ ENaC^+^ cells. Proximal tubular epithelial cells were defined as CD45^-^CD31^-^ENaC^-^ cells. Gating strategies for analyses of aortas and kidneys are depicted in S8 and S9 Figs, respectively.

### Histology

Kidneys were fixed in 4% formalin followed by paraffin embedding. 4 μm sections were sectioned and stained with Massons trichrome using standard protocols and the following reagents: Weigerts iron hematoxylin I and II (RH pharmacy, DK), Picric acid solution (Sigma), Briebrich Scarlet solution (Difco laboratories), wolframphoshporic acid hydrate (VWR) and methylene blue (Merck). The image analyses software Visiopharm (Visiopharm, Denmark) was used to measure glomerular diameter and determine kidney fibrosis. The glomerular diameter was defined as the widest part of the glomerulus and at least 25 glomeruli were measured per animal. Fibrosis was quantified automatically by Visiopharm as the blue stained area in Masson’s trichrome stained sections in the kidney cortex.

### Statistical analysis

Statistical analyses were performed using GraphPad Prism 6.00 (GraphPad Software Inc., San Diego). Comparisons were done with either Student's *t* test or one-way ANOVA followed by Sidak’s multiple comparisons post-test comparing SHAM with NX and NX with NX LIRA. *p* < 0.05 was considered significant.

## Supporting Information

S1 Fig Study outline. In Study 1 LDLr-/- mice underwent 5/6 nephrectomy (NX) or SHAM operation in a two-step procedure (week 1 and 3). Seven weeks later (week 10) the diet was changed from chow to a western type diet, and the study was terminated 9 weeks later (week 19). In Study 2 LDLr-/- mice underwent 5/6 nephrectomy (NX) or SHAM operation in a two-step procedure (week 1 and 3). Two weeks after the second operation (week 5) liraglutide treatment was initiated and two weeks later (week 7) the diet was changed from chow to a western type diet. After 11 weeks on the western type diet (week 18) the study was terminated. Blood samples were collected just before initiation of liraglutide treatment, just before the diet was changed, and eight weeks after initiation of liraglutide treatment).
(TIF)

S2 Fig The number of endothelial cells and distal tubular epithelial cells is reduced in kidneys from uremic LDLr-/- mice. Sixteen weeks after induction of uremia, flow cytometry was performed on kidneys from control (SHAM) and uremic (NX) LDLr-/- mice (n = 5 mice/group). The number of endothelial cells (**A**), distal tubular cells (DTECs; **B**) and proximal tubular cells (PTECs; **C**) relative to kidney weight is shown. Depicted values are mean±SEM. *p<0.05 as determined by unpaired students t-test. n = 5 mice per group.
(TIF)

S3 Fig The number of leukocytes is not affected in kidneys from uremic LDLr-/- mice. Sixteen weeks after induction of uremia, flow cytometry was performed on kidneys from control (SHAM) and uremic (NX) LDLr-/- mice (n = 5 mice/group). The number of leukocytes was analyzed by flow cytometry and normalized to kidney weight. Depicted values are mean±SEM. Statistics were made by unpaired students t-test.
(TIF)

S4 Fig Liraglutide treatment does not affect glucose levels in uremic mice. Glucose was measured in plasma from non-fasted animals treated with liraglutide for eight weeks. To obtain enough plasma for the measurement, plasma samples from two mice had to be pooled. A total of seven pools were measured per group (one sample was excluded from the NX LIRA group as the sample volume was too small). Depicted values are mean±SEM. Statistical analysis were made by 1-way ANOVA followed by Sidak’s multiple comparisons post-test.
(TIF)

S5 Fig Liraglutide treatment leads to a slight reduction in food intake in uremic mice. Accumulated food intake was measured by weighing the food every day and normalizing it to the number of mice in each cage. Depicted values are mean±SEM of 3 cages in each group.
(TIF)

S6 Fig Liraglutide does not affect immune cell composition or gene expression in aortas from uremic mice. A-D: flow cytometry analysis of aortas from control (SHAM; n = 6), uremic (NX; n = 6) and liraglutide treated uremic (NX LIRA; n = 6) LDLr-/- mice after 11 weeks of treatment with full dose liraglutide (1000 μg/kg) showing the number of leukocytes (**A**) and the myeloid (CD45^+^CD11b^+^) and lymphoid (CD45^+^CD11b^-^) cell distributions (**B**) as % of the leukocyte population. The mean fluorescence intensity of the M2 marker CD206 (**C**) and the M1 marker CD11c (**D**) was detected on myeloid cells. Depicted values are mean±SEM. Statistical analysis were made by 1-way ANOVA followed by Sidak’s multiple comparisons post-test.
(TIF)

S7 Fig NX leads to reduced levels of endothelial cells, proximal and distal tubular epithelial cells and there is no effect of liraglutide. Fifteen weeks after induction of uremia, flow cytometry was performed on kidneys from control (SHAM), uremic (NX), and liraglutide treated uremic (NX LIRA) LDLr-/- mice (n = 7 mice/group). The number of endothelial cells (**A**), distal tubular cells (**B**) and proximal tubular cells (**C**) relative to kidney weight are depicted. Depicted values are mean±SEM. *p<0.05, **p<0.01, ***p<0.005 as determined by 1-way ANOVA followed by Sidak’s multiple comparisons post-test.
(TIF)

S8 Fig The number of leukocytes in kidneys from NX mice tends to be reduced by liraglutide. Fifteen weeks after induction of uremia, flow cytometry was performed on kidneys from control (SHAM), uremic (NX), and liraglutide treated uremic (NX LIRA) LDLr-/- mice (n = 7 mice/group). The number of leukocytes in the kidney normalized to kidney weight is shown. Depicted values are mean±SEM. *p<0.05, **p<0.01, ***p<0.005 as determined by 1-way ANOVA followed by Sidak’s multiple comparisons post-test.
(TIF)

S9 Fig Gating strategy for flow cytometry analysis of aortas. Cells were first gated according to the forward and sideward scatter followed by detection of CD45^+^ cells in a CD45/Galectin-3 plot and adjusted in a FCS-A/SSC-A backgate. Galectin-3 was included as a macrophage activation marker and used here for optimal separation between populations, but not quantified (data not shown). Finally lymphoid and myeloid cells were gated in a CD45/CD11b plot.
(TIF)

S10 Fig Gating strategy for flow cytometry analysis of kidney cells. Kidney flow analysis was performed using 3 antibody panels, detecting endothelial cells, DTECs and PTECs (**A**), CD4^+^ and CD8^+^ T-cells (**B**) and monocyte-like and macrophage-like cells (**C**). In **A**, cells were first gated according to the forward and sideward scatter. Then duplicates were removed in first a FSC-H/FSC-W gate and then a SSC-H/SSC-W followed by removal of dead cells in a 7AAD/SCC-A plot. CD45^+^ cells were subsequently detected and excluded from further analysis in a CD45^+^/SSC-A plot. The remaining cells were then gated for endothelial cells in a CD31^+^/SSC-A plot followed by detection of ENAC^+^ cells in the CD45^-^CD31^-^ population in a FSC-A/ENAC^+^. In **B**, cells were first gated according to the forward and sideward scatter. Then duplicates were removed in first a FSC-H/FSC-W gate, and then a SSC-H/SSC-W followed by removal of dead cells in a 7AAD/FSC-A plot. CD45^+^ cells were subsequently determined by a CD45^+^/SSC-A plot and adjusted in a FCS-A/SSC-A backgate. Finally CD4^+^ and CD8^+^ cells were gated in a CD4/CD8 plot. In **C**, cells were first gated according to the forward and sideward scatter followed by removal of duplicates in first a FSC-H/FSC-W gate and then a SSC-H/SSC-W. CD45^+^ cells were then detected in a CD45^+^/FSC-A plot and adjusted in a FCS-A/SSC-A backgate. Finally a CD68/F4/80 plot was used to detect single positive CD68^+^F4/80^-^ and double positive CD68^+^F4/80^-^ cells.
(TIF)

S1 Table Plasma biochemistry data for study 1. (PDF)

S2 Table Plasma biochemistry data for study 2. (PDF)

S3 Table NX leads to increased plasma cytokine levels, and there is no effect of liraglutide. Plasma cytokine levels measured at termination of Study 2 are depicted as fold change of uremic (NX) mice relative to control (SHAM) mice (black bars) and liraglutide treated uremic mice (NX LIRA) relative to uremic (NX) mice (grey bars). Depicted values are mean±SEM. Statistical analysis was made by 1-way ANOVA followed by Sidak’s multiple comparisons post-test. *p<0.05, **p<0.01, ***p<0.005, ****p<0.001. n = 14–15 mice per group. OPN: Osteopontin, MCP-1: Monocyte Chemoattractant Protein-1, TNFα: Tumor necrosis factor alpha, KC: chemokine (C-X-C motif) ligand 1, IL-6: interleukin 6, IFNγ: interferon gamma, IL-10: interleukin 10, IL-5: interleukin 5.
(PDF)

S4 Table Primer ID list. (PDF)
