# Supplementary figures and images for: Liraglutide Reduces Both Atherosclerosis and Kidney Inflammation in Moderately Uremic LDLr-/- Mice
Source: PLoS One. 2016 Dec 16;11(12):e0168396. doi: 10.1371/journal.pone.0168396 (PMC5161477; doi:10.1371/journal.pone.0168396)

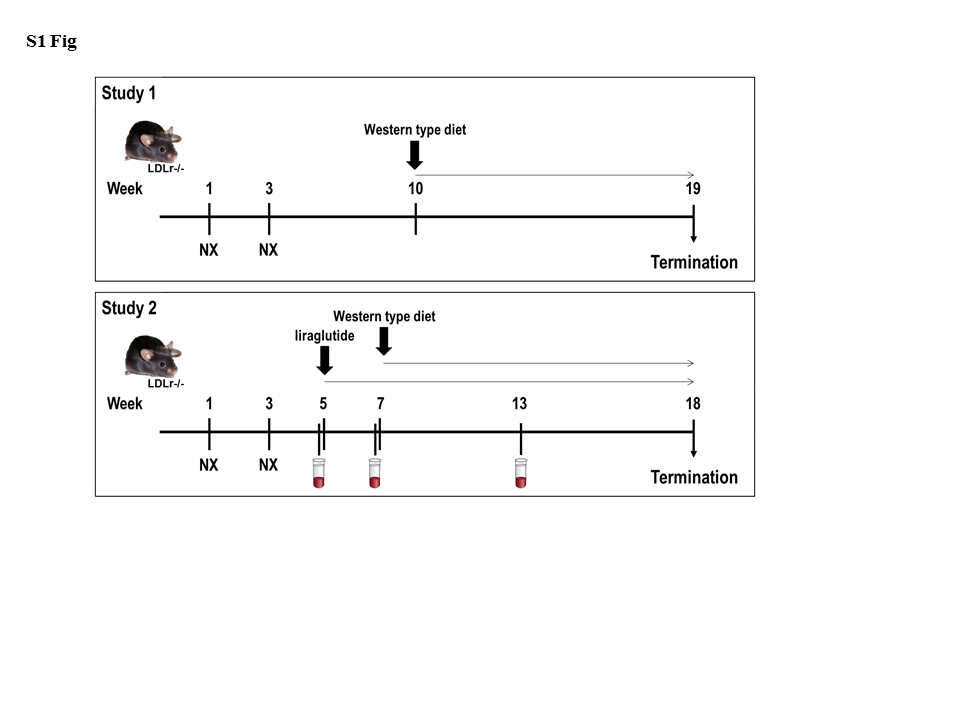

Supplement: S1 Fig — In Study 1 LDLr-/- mice underwent 5/6 nephrectomy (NX) or SHAM operation in a two-step procedure (week 1 and 3). Seven weeks later (week 10) the diet was changed from chow to a western type diet, and the study was terminated 9 weeks later (week 19). In Study 2 LDLr-/- mice underwent 5/6 nephrectomy (NX) or SHAM operation in a two-step procedure (week 1 and 3). Two weeks after the second operation (week 5) liraglutide treatment was initiated and two weeks later (week 7) the diet was changed from chow to a western type diet. After 11 weeks on the western type diet (week 18) the study was terminated. Blood samples were collected just before initiation of liraglutide treatment, just before the diet was changed, and eight weeks after initiation of liraglutide treatment). (TIF) [file pone.0168396.s001.TIF]

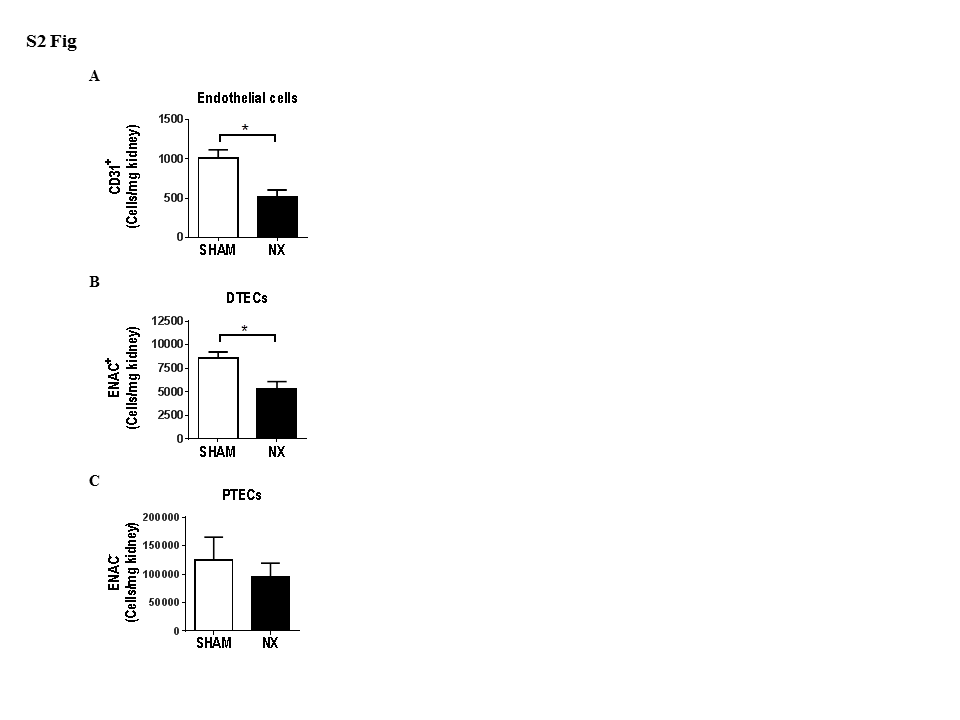

Supplement: S2 Fig — Sixteen weeks after induction of uremia, flow cytometry was performed on kidneys from control (SHAM) and uremic (NX) LDLr-/- mice (n = 5 mice/group). The number of endothelial cells (A), distal tubular cells (DTECs; B) and proximal tubular cells (PTECs; C) relative to kidney weight is shown. Depicted values are mean±SEM. *p<0.05 as determined by unpaired students t-test. n = 5 mice per group. (TIF) [file pone.0168396.s002.TIF]

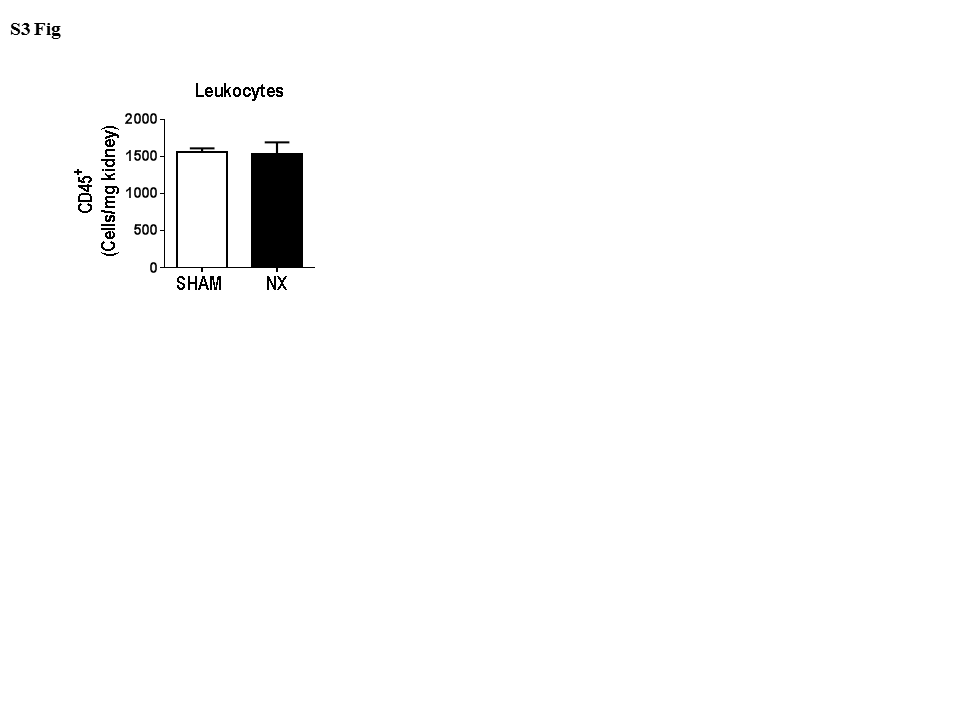

Supplement: S3 Fig — Sixteen weeks after induction of uremia, flow cytometry was performed on kidneys from control (SHAM) and uremic (NX) LDLr-/- mice (n = 5 mice/group). The number of leukocytes was analyzed by flow cytometry and normalized to kidney weight. Depicted values are mean±SEM. Statistics were made by unpaired students t-test. (TIF) [file pone.0168396.s003.TIF]

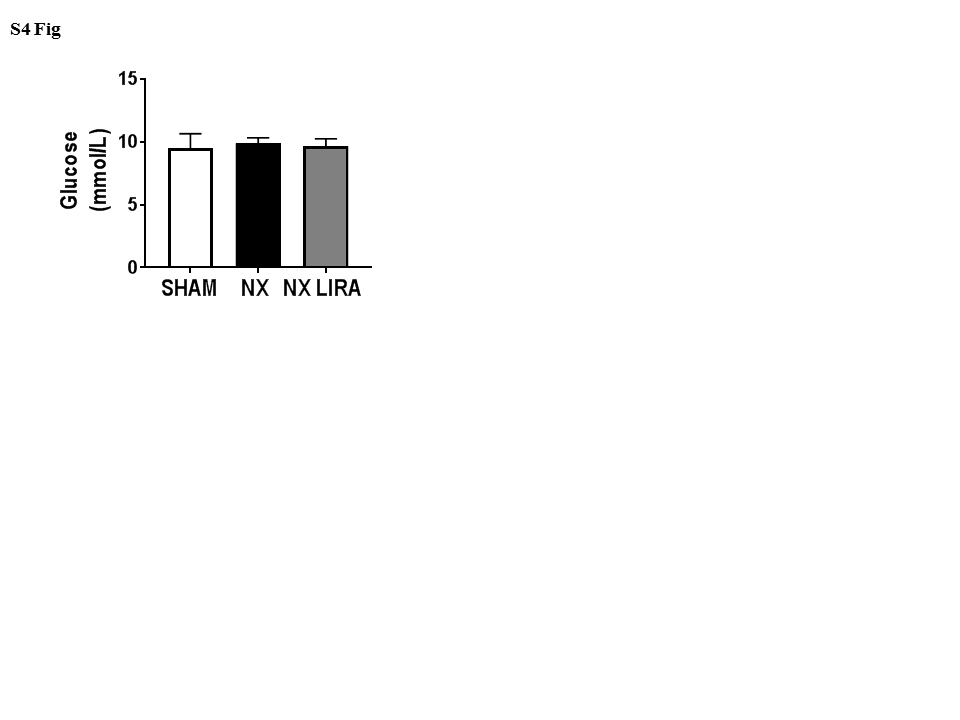

Supplement: S4 Fig — Glucose was measured in plasma from non-fasted animals treated with liraglutide for eight weeks. To obtain enough plasma for the measurement, plasma samples from two mice had to be pooled. A total of seven pools were measured per group (one sample was excluded from the NX LIRA group as the sample volume was too small). Depicted values are mean±SEM. Statistical analysis were made by 1-way ANOVA followed by Sidak’s multiple comparisons post-test. (TIF) [file pone.0168396.s004.TIF]

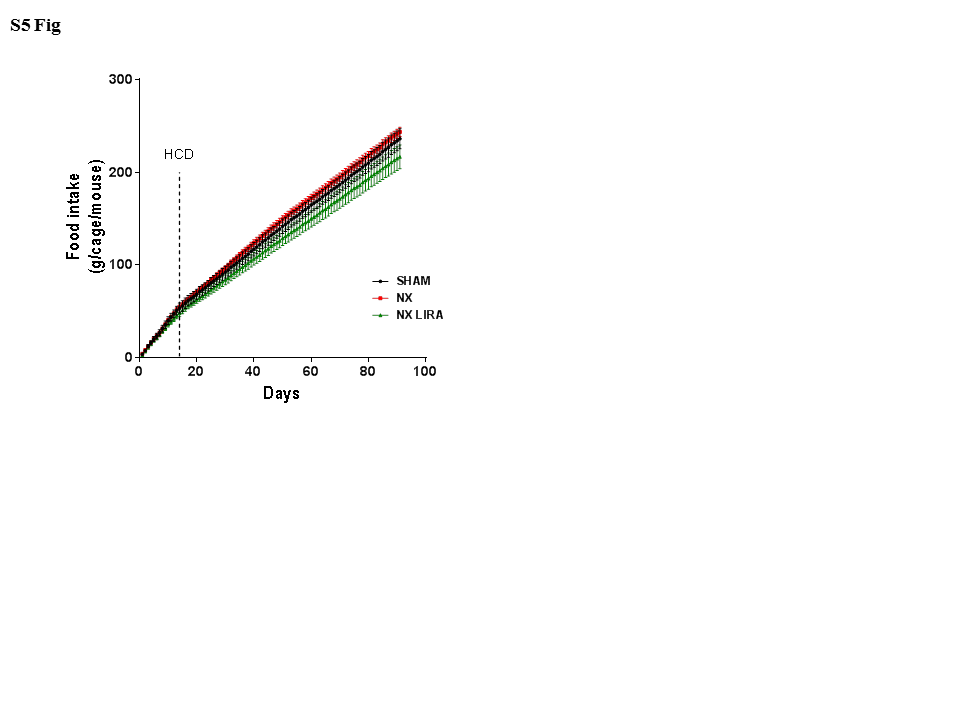

Supplement: S5 Fig — Accumulated food intake was measured by weighing the food every day and normalizing it to the number of mice in each cage. Depicted values are mean±SEM of 3 cages in each group. (TIF) [file pone.0168396.s005.TIF]

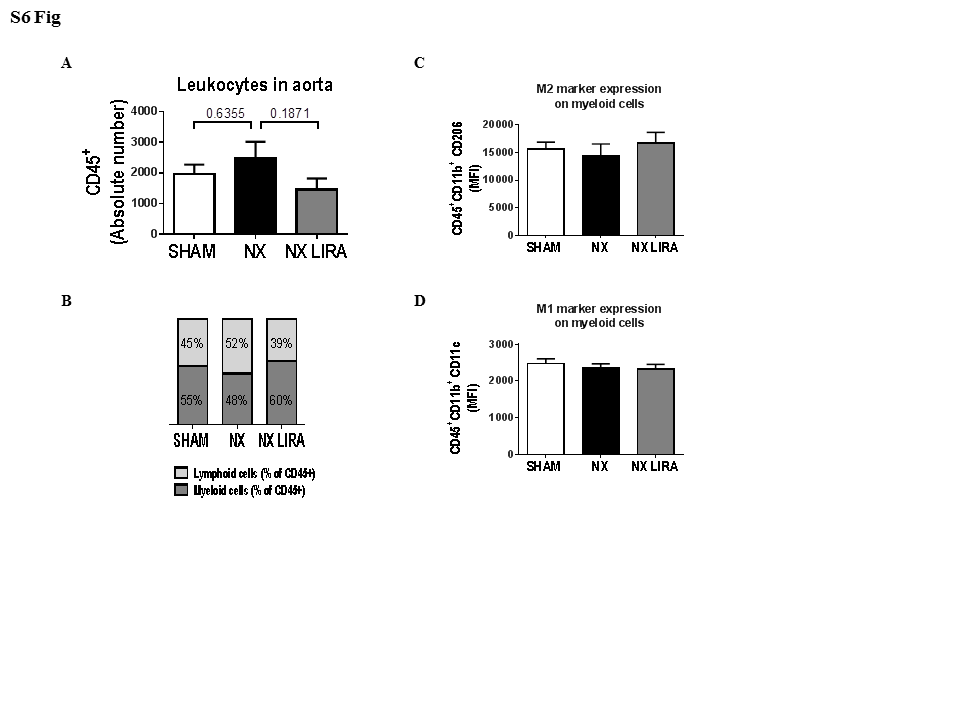

Supplement: S6 Fig — A-D: flow cytometry analysis of aortas from control (SHAM; n = 6), uremic (NX; n = 6) and liraglutide treated uremic (NX LIRA; n = 6) LDLr-/- mice after 11 weeks of treatment with full dose liraglutide (1000 μg/kg) showing the number of leukocytes (A) and the myeloid (CD45+CD11b+) and lymphoid (CD45+CD11b-) cell distributions (B) as % of the leukocyte population. The mean fluorescence intensity of the M2 marker CD206 (C) and the M1 marker CD11c (D) was detected on myeloid cells. Depicted values are mean±SEM. Statistical analysis were made by 1-way ANOVA followed by Sidak’s multiple comparisons post-test. (TIF) [file pone.0168396.s006.TIF]

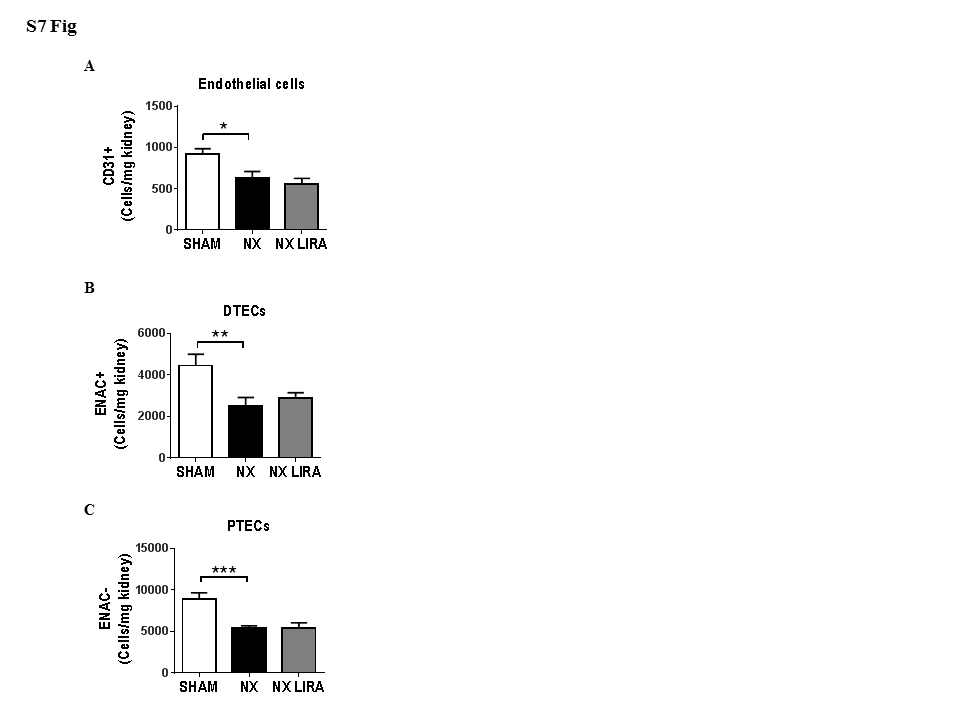

Supplement: S7 Fig — Fifteen weeks after induction of uremia, flow cytometry was performed on kidneys from control (SHAM), uremic (NX), and liraglutide treated uremic (NX LIRA) LDLr-/- mice (n = 7 mice/group). The number of endothelial cells (A), distal tubular cells (B) and proximal tubular cells (C) relative to kidney weight are depicted. Depicted values are mean±SEM. *p<0.05, **p<0.01, ***p<0.005 as determined by 1-way ANOVA followed by Sidak’s multiple comparisons post-test. (TIF) [file pone.0168396.s007.TIF]

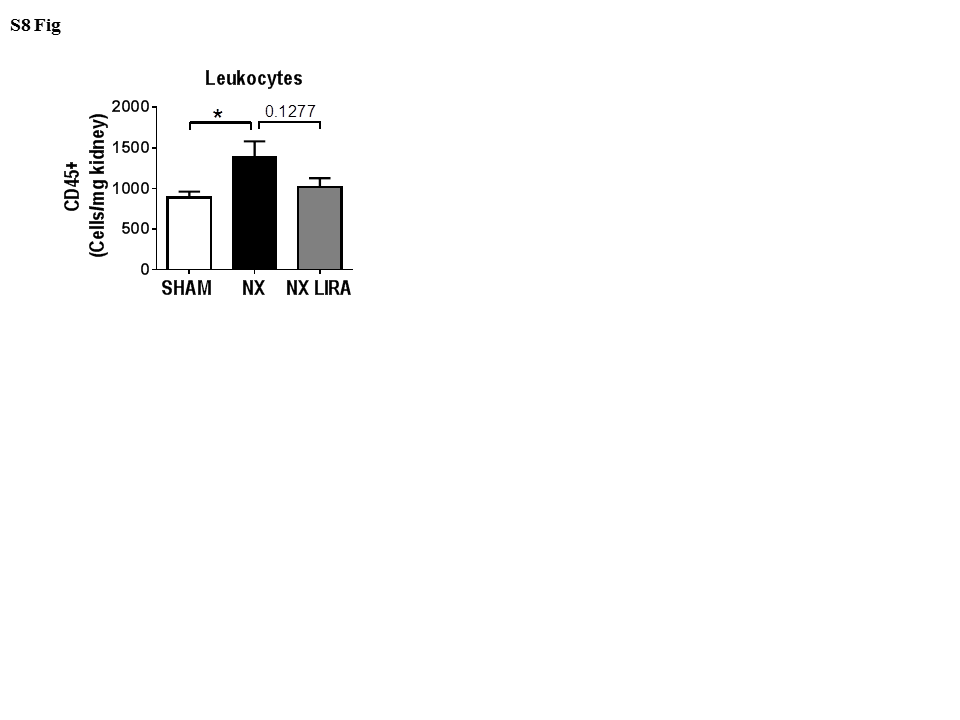

Supplement: S8 Fig — Fifteen weeks after induction of uremia, flow cytometry was performed on kidneys from control (SHAM), uremic (NX), and liraglutide treated uremic (NX LIRA) LDLr-/- mice (n = 7 mice/group). The number of leukocytes in the kidney normalized to kidney weight is shown. Depicted values are mean±SEM. *p<0.05, **p<0.01, ***p<0.005 as determined by 1-way ANOVA followed by Sidak’s multiple comparisons post-test. (TIF) [file pone.0168396.s008.TIF]

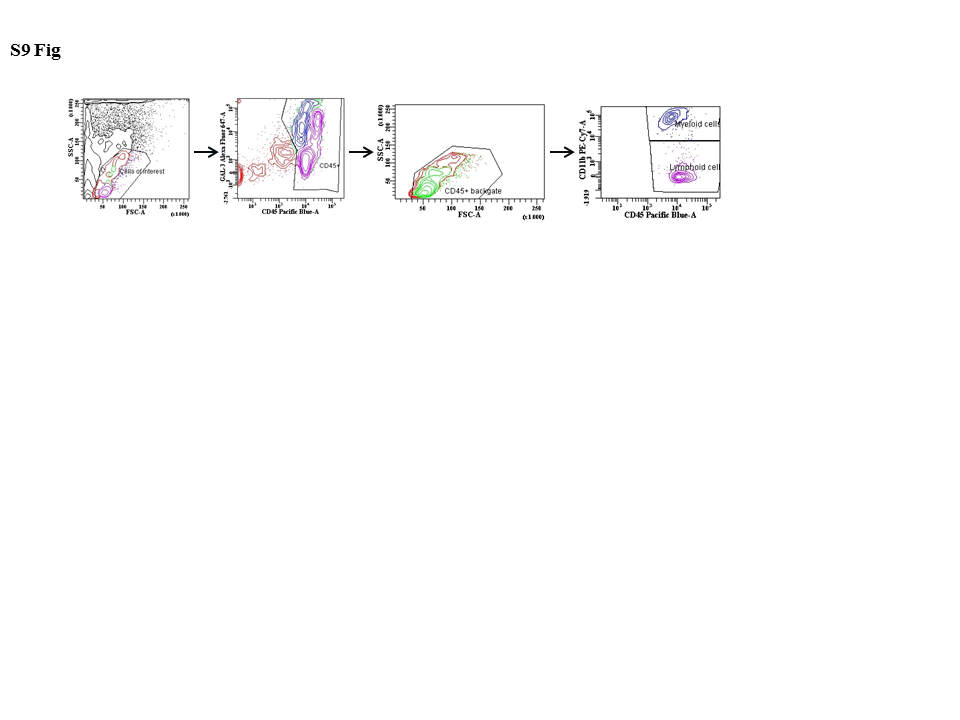

Supplement: S9 Fig — Cells were first gated according to the forward and sideward scatter followed by detection of CD45+ cells in a CD45/Galectin-3 plot and adjusted in a FCS-A/SSC-A backgate. Galectin-3 was included as a macrophage activation marker and used here for optimal separation between populations, but not quantified (data not shown). Finally lymphoid and myeloid cells were gated in a CD45/CD11b plot. (TIF) [file pone.0168396.s009.TIF]

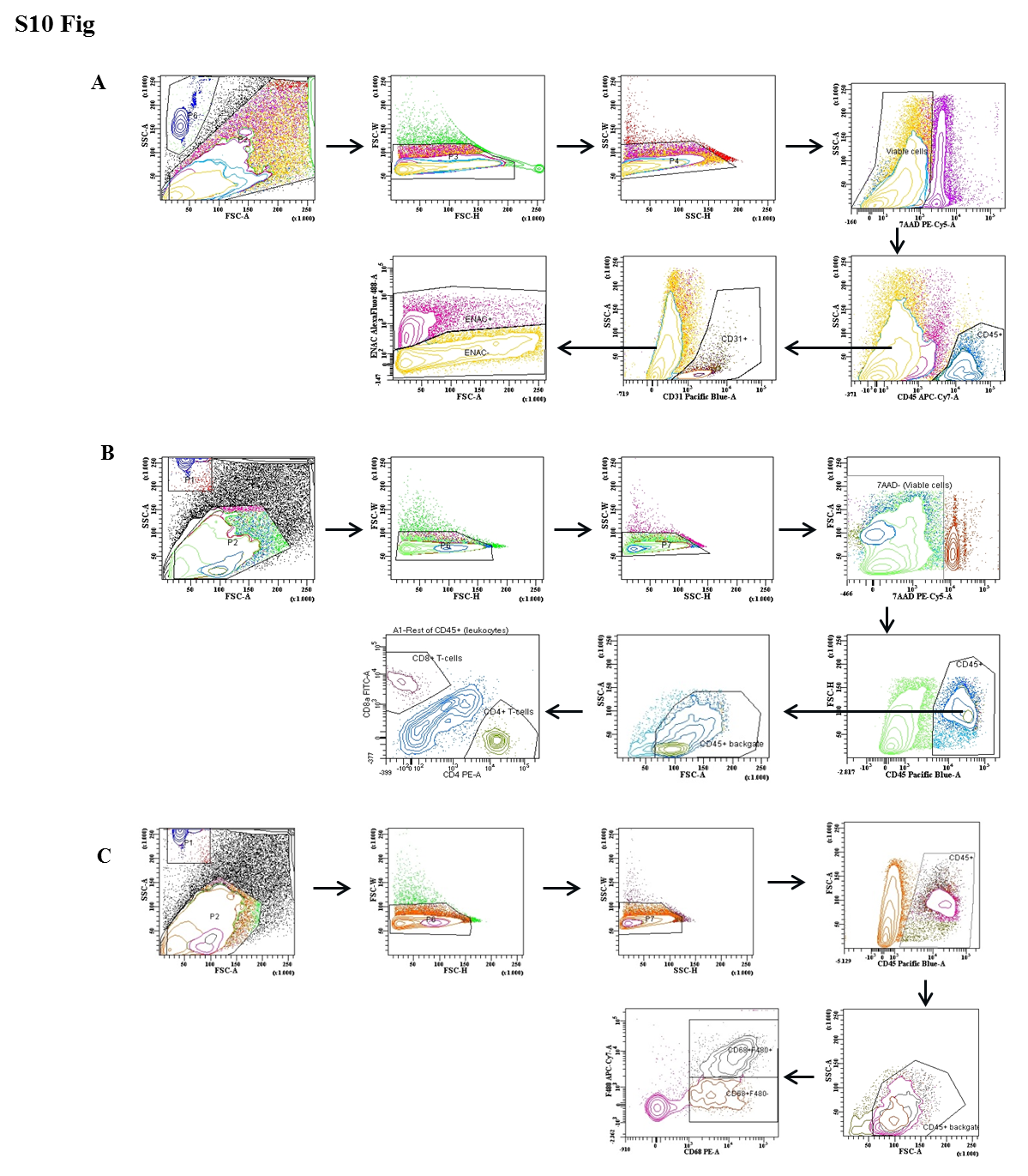

Supplement: S10 Fig — Kidney flow analysis was performed using 3 antibody panels, detecting endothelial cells, DTECs and PTECs (A), CD4+ and CD8+ T-cells (B) and monocyte-like and macrophage-like cells (C). In A, cells were first gated according to the forward and sideward scatter. Then duplicates were removed in first a FSC-H/FSC-W gate and then a SSC-H/SSC-W followed by removal of dead cells in a 7AAD/SCC-A plot. CD45+ cells were subsequently detected and excluded from further analysis in a CD45+/SSC-A plot. The remaining cells were then gated for endothelial cells in a CD31+/SSC-A plot followed by detection of ENAC+ cells in the CD45-CD31- population in a FSC-A/ENAC+. In B, cells were first gated according to the forward and sideward scatter. Then duplicates were removed in first a FSC-H/FSC-W gate, and then a SSC-H/SSC-W followed by removal of dead cells in a 7AAD/FSC-A plot. CD45+ cells were subsequently determined by a CD45+/SSC-A plot and adjusted in a FCS-A/SSC-A backgate. Finally CD4+ and CD8+ cells were gated in a CD4/CD8 plot. In C, cells were first gated according to the forward and sideward scatter followed by removal of duplicates in first a FSC-H/FSC-W gate and then a SSC-H/SSC-W. CD45+ cells were then detected in a CD45+/FSC-A plot and adjusted in a FCS-A/SSC-A backgate. Finally a CD68/F4/80 plot was used to detect single positive CD68+F4/80- and double positive CD68+F4/80- cells. (TIF) [file pone.0168396.s010.tif]
