# Supplementary material for: Liraglutide Reduces Both Atherosclerosis and Kidney Inflammation in Moderately Uremic LDLr-/- Mice
Source: PLoS One. 2016 Dec 16;11(12):e0168396. doi: 10.1371/journal.pone.0168396 (PMC5161477; doi:10.1371/journal.pone.0168396)
Supplement: S1 Table — (PDF) [file pone.0168396.s011.pdf]

S1 Table

	SHAM			NX			
N	15			17			
Body weight (g)	23.3	±	0.3	22.1	±	0.4	
P-urea (mmol/L)	8.1	±	0.4	19.6	±	1.0	****
P-creatinine (µmol/L)	14.8	±	1.0	23.9	±	0.8	****
P-cholesterol (mmol/L)	31.7	±	1.7	34.1	±	2.1	
P-phospate	2.3	±	0.1	2.4	±	0.2	
P-Ca^{2+} (mmol/L)	2.2	±	0.02	2.6	±	0.04	****
P-osteopontin	207.4	±	11.9	437.9	±	25.2	****

Results are depicted as mean±SEM. ****P< 0.0001 as determined by unpaired students t-test.
