# Supplementary material for: Liraglutide Reduces Both Atherosclerosis and Kidney Inflammation in Moderately Uremic LDLr-/- Mice
Source: PLoS One. 2016 Dec 16;11(12):e0168396. doi: 10.1371/journal.pone.0168396 (PMC5161477; doi:10.1371/journal.pone.0168396)
Supplement: S2 Table — (PDF) [file pone.0168396.s012.pdf]

S2 Table

	SHAM	NX	NX LIRA
N	14	14	15
**Lira-treatment baseline**			
Body weight (g)	20.4 ± 0.3	20.3 ± 0.5	19.8 ± 0.3
P-urea (mmol/L)	10.3 ± 0.3	30.5 ± 2.6 [a]	27.6 ± 2.3
P-cholesterol (mmol/L)	6.5 ± 0.2	7.7 ± 0.2 [b]	7.3 ± 0.2
P-phospate	1.9 ± 0.08	1.8 ± 0.05	1.8 ± 0.08
**Termination**			
Body weight (g)	21.7 ± 0.3	21.2 ± 0.2	19.8 ± 0.3 [c]
P-urea (mmol/L)	9.4 ± 0.4	20.5 ± 1.4 [a]	19.9 ± 1.6
P-creatinine (µmol/L)	15.3 ± 1.3	24.1 ± 1.2 [a]	20.6 ± 1.2
P-cholesterol (mmol/L)	40.2 ± 1.3	40.7 ± 1.6	41.9 ± 2.7
P-phospate	1.8 ± 0.09	1.9 ± 0.09	1.9 ± 0.11
P- Ca^{2+} (mmol/L)	2.2 ± 0.02	2.5 ± 0.03 [a]	2.5 ± 0.04

Results are depicted as mean±SEM. Statistical analysis was determined by 1-way ANOVA followed by Sidak’s multiple comparisons post-test.

[a]=**** (SHAM vs NX)

[b]=*** (SHAM vs NX)

[c]=** (NX vs NX LIRA)
