# Supplementary material for: Liraglutide Reduces Both Atherosclerosis and Kidney Inflammation in Moderately Uremic LDLr-/- Mice
Source: PLoS One. 2016 Dec 16;11(12):e0168396. doi: 10.1371/journal.pone.0168396 (PMC5161477; doi:10.1371/journal.pone.0168396)
Supplement: S3 Table — Plasma cytokine levels measured at termination of Study 2 are depicted as fold change of uremic (NX) mice relative to control (SHAM) mice (black bars) and liraglutide treated uremic mice (NX LIRA) relative to uremic (NX) mice (grey bars). Depicted values are mean±SEM. Statistical analysis was made by 1-way ANOVA followed by Sidak’s multiple comparisons post-test. *p<0.05, **p<0.01, ***p<0.005, ****p<0.001. n = 14–15 mice per group. OPN: Osteopontin, MCP-1: Monocyte Chemoattractant Protein-1, TNFα: Tumor necrosis factor alpha, KC: chemokine (C-X-C motif) ligand 1, IL-6: interleukin 6, IFNγ: interferon gamma, IL-10: interleukin 10, IL-5: interleukin 5. (PDF) [file pone.0168396.s013.pdf]

S3 Table

Cytokine	SHAM	NX	NX LIRA	NX rel. to SHAM mean fold change		NX LIRA rel. to NX mean fold change
IFNγ	0.34 ± 0.06	0.85 ± 0.14	0.85 ± 0.08	2.50 ± 0.41	**	1.01 ± 0.09
IL-10	20.90 ± 2.83	43.76 ± 4.64	33.12 ± 3.17	2.09 ± 0.22	***	0.76 ± 0.72
IL-5	2.45 ± 0.24	4.00 ± 0.41	5.82 ± 0.99	1.63 ± 0.17		1.45 ± 0.25
IL-6	23.28 ± 2.85	37.53 ± 13.03	54.02 ± 14.42	1.61 ± 0.56		1.44 ± 0.38
KC	153.80 ± 13.38	144.40 ± 11.48	165.70 ± 13.9	0.94 ± 0.07		1.15 ± 0.10
TNFα	24.36 ± 1.92	47.30 ± 3.55	44.07 ± 3.73	1.94 ± 0.15	****	0.93 ± 0.08
MCP-1	983.10 ± 59.44	825.20 ± 53.01	968.40 ± 90.05	0.84 ± 0.05		1.17 ± 0.11
OPN	252.30 ± 18.53	396.00 ± 23.87	391.00 ± 14.51	1.57 ± 0.09	*	0.99 ± 0.04

Plasma cytokine levels measured at termination of Study 2 are shown as absolute values in column 1-3 (pg/mL, OPN: ng/mL) as well as fold change of uremic (NX) mice relative to control (SHAM) mice (column 4) and liraglutide treated uremic mice (NX LIRA) relative to uremic (NX) mice (column 5). Depicted values are mean±SEM. Statistical analysis was made by 1-way ANOVA followed by Sidak's multiple comparisons post-test. *p<0.05, **p<0.01, ***p<0.005, ****p<0.001. n=14-15 mice per group. OPN: Osteopontin, MCP-1: Monocyte Chemoattractant Protein-1, TNFα: Tumor necrosis factor alpha, KC: chemokine (C-X-C motif) ligand 1, IL-6: interleukin 6, IFNɣ: interferon gamma, IL-10: interleukin 10, IL-5: interleukin 5.
