# Supplementary material for: Liraglutide Reduces Both Atherosclerosis and Kidney Inflammation in Moderately Uremic LDLr-/- Mice
Source: PLoS One. 2016 Dec 16;11(12):e0168396. doi: 10.1371/journal.pone.0168396 (PMC5161477; doi:10.1371/journal.pone.0168396)
Supplement: S4 Table — (PDF) [file pone.0168396.s014.pdf]

S4 Table

Gene name	Assay ID
Ace	Mm00802048_m1
Ager	Mm01134790_g1
Agt	Mm00599662_m1
Ccl12	Mm01617100_m1
Ccl2	Mm00441242_m1
Ccl5	Mm01302427_m1
Cdh1	Mm01247357_m1
Cdh5	Mm00486938_m1
Cdkn1a	Mm04205640_g1
Col1a1	Mm00801666_g1
Col1a2	Mm00483888_m1
Col3a1	Mm01254476_m1
Ctgf	Mm01192932_g1
Cxcl15	Mm00441263_m1
Fn1	Mm01256744_m1
Grem1	Mm00488615_s1
Havcr1	Mm00506686_m1
Hif1a	Mm00468869_m1
Hspg2	Mm01181173_g1
Icam1	Mm00516023_m1
Itga1	Mm01306375_m1
Itga3	Mm00442910_m1
Itga5	Mm00439797_m1
Kcp	Mm01159615_m1
Lamc1	Mm00711820_m1
Lcn2	Mm01324470_m1
Mmp2	Mm00439498_m1
Nos2	Mm00440502_m1
Nos3	Mm00435217_m1
Nphs1	Mm00497828_m1
Pdgfb	Mm00440677_m1
Pdgfrb	Mm00435546_m1
Serpine1	Mm00435860_m1
Snai1	Mm00441533_g1
Tgfb1	Mm01178820_m1
Tgfb2	Mm00436955_m1
Tgfb3	Mm00436960_m1
Timp1	Mm00441818_m1
Timp2	Mm00441825_m1
Tlr2	Mm00442346_m1
Tnf	Mm00443258_m1
Vcam1	Mm01320970_m1
Vim	Mm01333430_m1
Wt1	Mm00460570_m1
Rpl27	Mm01245874_g1
Rps13	Mm00850011_g1
Ubc	Mm02525934_g1

Primer ID list
